# Effects of Treadmill Exercise on Neural Mitochondrial Functions in Parkinson’s Disease: A Systematic Review of Animal Studies

**DOI:** 10.3390/biomedicines9081011

**Published:** 2021-08-13

**Authors:** Nguyen Thanh Nhu, Yu-Jung Cheng, Shin-Da Lee

**Affiliations:** 1 Faculty of Medicine, Can Tho University of Medicine and Pharmacy, Can Tho 94117, Vietnam; ntnhu@ctump.edu.vn; 2 Department of Physical Therapy, Graduate Institute of Rehabilitation Science, China Medical University, Taichung 41354, Taiwan; chengyu@mail.cmu.edu.tw; 3 School of Rehabilitation Medicine, Weifang Medical University, Weifang 261053, China; 4 Department of Physical Therapy, Asia University, Taichung 41354, Taiwan

**Keywords:** treadmill exercise, Parkinson’s disease, neural mitochondrial functions

## Abstract

This systematic review sought to determine the effects of treadmill exercise on the neural mitochondrial respiratory deficiency and neural mitochondrial quality-control dysregulation in Parkinson’s disease. PubMed, Web of Science, and EMBASE databases were searched through March 2020. The English-published animal studies that mentioned the effects of treadmill exercise on neural mitochondria in Parkinson’s disease were included. The CAMARADES checklist was used to assess the methodological quality of the studies. Ten controlled trials were included (median CAMARADES score = 5.7/10) with various treadmill exercise durations (1–18 weeks). Seven studies analyzed the neural mitochondrial respiration, showing that treadmill training attenuated complex I deficits, cytochrome c release, ATP depletion, and complexes II–V abnormalities in Parkinson’s disease. Nine studies analyzed the neural mitochondrial quality-control, reporting that treadmill exercise improved mitochondrial biogenesis, mitochondrial fusion, and mitophagy in Parkinson’s disease. The review findings supported the hypothesis that treadmill training could attenuate both neural mitochondrial respiratory deficiency and neural mitochondrial quality-control dysregulation in Parkinson’s disease, suggesting that treadmill training might slow down the progression of Parkinson’s disease.

## 1. Introduction

Parkinson’s disease (PD) is the second most common neurodegenerative disorder, causing a considerable number of disabilities globally [1,2]. The underlying mechanisms of PD are unclear, making it difficult to find efficient targeted therapies [3]. The current treatment of PD addresses symptomatic improvement only because none of the available treatment strategies have been confirmed to slow down PD progression [2].

Recently, neural mitochondrial respiratory deficiency has emerged as a central hallmark of Parkinsonian etiology [4,5]. In PD, the electron transport system of the neural mitochondria is impaired, mainly characterized by mitochondrial complex I deficit, cytochrome *c* release, and ATP depletion [6]. Impaired mitochondrial respiration in the brain increases oxidative stress and neuron loss, thereby augmenting PD progression [4].

Studies have also linked neural mitochondrial respiratory deficiency to neural mitochondrial quality-control dysregulation in PD [7,8]. In the physiological condition, neural mitochondrial quality-control involves a balance among biogenesis, dynamics (fusion/fission), and mitophagy (autophagy of mitochondria) [7]. Mitochondrial biogenesis produces the new mitochondria and mitochondrial content, accompanied by mitochondrial fusion to maintain a healthy mitochondrial network [9]. Meanwhile, mitochondrial fission segregates the damaged mitochondria and provides those for the mitophagy process, preventing the accumulation of dysfunctional mitochondria in the brain [10].

In PD, the biogenesis regulators and import machinery of neural mitochondria are reduced, leading to inhibition of mitochondrial biogenesis [11]. Additionally, PD has been confirmed to induce an imbalance of neural mitochondrial dynamics (fusion/fission) [4]. Moreover, the mitophagy process has been proven to be impaired with reductions of lysosomal activities in PD [12]. Disorders of biogenesis, fusion/fission, and mitophagy reduce the quantity and quality of neural mitochondria, leading to a neural mitochondrial respiratory deficiency in PD [4,7].

Treadmill exercise (TE) has been widely applied in PD rehabilitation [13,14]. Previous evidence indicated that TE training improved both the symptoms and the quality of life in PD patients [13]. In addition, a previous study showed that TE training improved gait functions by modulating neural mitochondrial dynamics in a PD rats model [15]. Another study reported that TE training reduced both neuron loss and behavioral disorders by improving mitochondrial respiration in a PD mouse model [16]. Those data suggested that TE training not only improves symptoms but also delays PD progression by attenuating PD-induced neural mitochondrial damage. However, the various approaches of the individual studies make it difficult to comprehensively understand the effects of TE on neural mitochondria in PD. Although several systematic reviews have been carried out to summarize the neuroprotective effects of TE training on PD, none of them specifically analyzed neural mitochondrial respiratory deficiency and neural mitochondrial quality-control dysregulation.

Therefore, we conducted a systematic review of animal studies to summarize the effects of TE training on mitochondrial functions in PD, focusing on the following objectives: (1) The effects of TE training on neural mitochondrial respiratory deficiency in PD; and (2) The effects of TE training on neural mitochondrial quality-control dysregulation in PD, including neural mitochondrial biogenesis, neural mitochondrial dynamics (fusion/fission), and neural mitophagy.

## 2. Materials and Methods

### 2.1. Protocol and Registration

The protocol of this systematic review was registered on PROSPERO with the registration number: CRD42020164122. We followed “the Preferred Reporting Items for Systematic Reviews and Meta-Analyses (PRISMA) checklist” [17] and “the PRISMA for abstract checklist” [18] to conduct and report this systematic review.

### 2.2. Eligibility Criteria

*Types of study designs:* controlled-trial animal studies with separate experimental groups. The studies were English publications without any restriction of publication date. Protocol articles, case reports, reviews, and conference abstracts were excluded from this systematic review.

*Types of animal models:* animal models of PD. Sex, age, and species of the subjects were not restricted. Studies were excluded if they did not provide sufficient data about the animal species or the PD induction (model types and timing).

*Types of intervention:* treadmill exercise (TE) training without any restriction of the protocol. The information about timing, duration, and frequency must be provided. Studies that evaluated the effects of TE in combination with the other therapies were excluded.

*Type of comparators:* studies that at least reported a comparison among a normal group, a sedentary PD group, and a TE-trained PD group. The normal control group must not be treated with any other therapeutic methods.

*Type of outcomes:* for the effects of TE training on neural mitochondrial respiratory deficiency in PD (the first objective), our outcomes were the components of the electron transport system (complexes I–V, cytochrome c, coenzyme Q10) and ATP production. For the effects of TE training on neural mitochondrial quality-control dysregulation in PD (the second objective), our outcomes included neural mitochondrial biogenesis, neural mitochondrial dynamics (fusion/fission), and neural mitophagy. The neural mitochondrial biogenesis outcome was measured through biogenesis regulators and translocase factors. The neural mitochondrial dynamic outcome was measured through fusion factors and fission factors. The neural mitophagy outcome was measured through dysfunctional mitochondria detectors, autophagosomal factors, and lysosomal factors.

### 2.3. Information Sources and Search Strategy

Relevant studies were identified by keywords searching on PubMed, Web of Science and EMBASE databases through March 2020, with a combination of the terms: (*“Parkinson” OR “Parkinsonism” OR “Parkinsonian”*) *AND “treadmill” AND* (“*mitochondria*” *OR* “*mitochondrial*” *OR* “*mitochondrion*” *OR* “*mitophagy*” *OR* “*ATP*” *OR* “*SIRT*” *OR* “*AMPK*” *OR* “*PGC-1α*” *OR* “*TFAM*” *OR* “*NRF*” *OR* “*mito-fusion*” *OR* “*mito-fission*”). We also reviewed references of the included studies to find other eligible papers. Briefly, the titles and abstracts of studies were screened to exclude duplications and irrelevant studies that did not mention the relevant information of Parkinson’s disease and exercise in their abstracts. Then, full texts were assessed and read to see if they met the eligibility criteria. Two independent assessors conducted the process of study selection. When a disagreement occurred, two assessors discussed with a third consultant to make the final decision.

### 2.4. Data Collection Process

Two independent reviewers extracted the data (including study characteristics and outcomes) by reading the text, graphs, and tables of the included studies. If the data were not available, we contacted the corresponding authors to request it. For study characteristics, we extracted the data of the first author’s name, published year, PD model (type, species, and sex), and TE protocol (timing, frequency, duration, and speed). For the outcome extraction, we extracted the data of neural mitochondrial respiration, neural mitochondrial biogenesis, neural mitochondrial dynamics (fusion/fission), and neural mitophagy.

### 2.5. Study Quality Evaluation

The study quality was evaluated by using the “Collaborative Approach to Meta-Analysis and Review of Animal Data from Experimental Studies” (CAMARADES) checklist with ten items [19]. Two authors independently evaluated and filled in the predesigned datasheets of the CAMARADES checklist, then disagreements were resolved by discussing among the two evaluators and the third consultant.

### 2.6. Data Synthesis and Presentation

The results of the search strategy are shown in the PRISMA flowchart and the narrative synthesis. Text and tables were used to present the study characteristics and the outcomes. For presentation of study characteristics, the summaries of the PD models, TE protocols, and types of outcomes are provided. For presentation of the outcomes, the effects of TE on neural mitochondrial respiratory deficiency and mitochondrial quality-control dysregulation in PD are described in the comparison among the normal control group, the PD group, and the TE-trained PD group.

## 3. Results

### 3.1. Search Results

**Figure 1:**
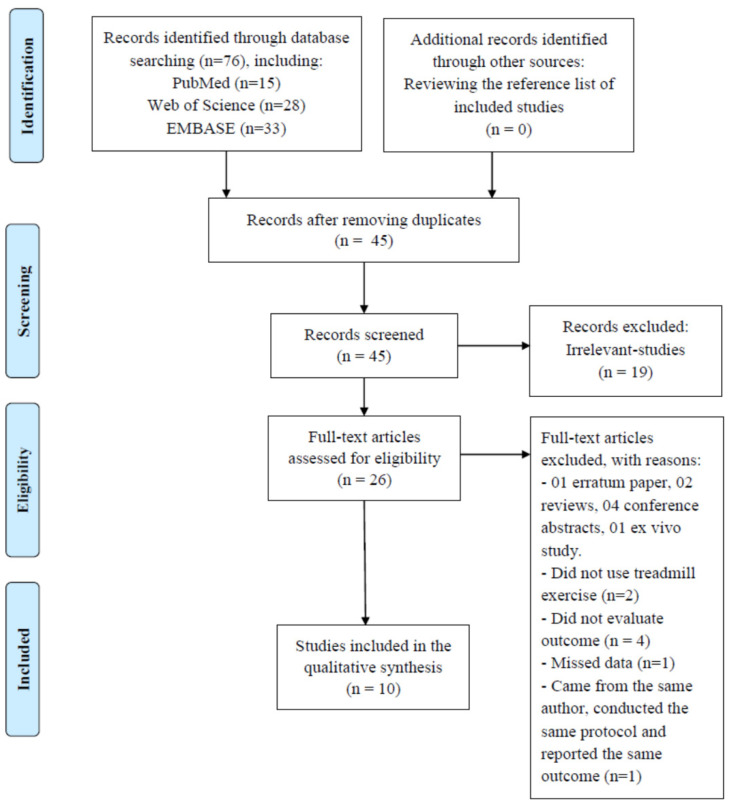
PRISMA flow chart for the selected protocol.

A total of 76 articles were found from PubMed (n = 15), Web of Science (n = 28), and EMBASE (n = 33) (Figure 1). After title and abstract screening, we removed 50 records: 31 duplications and 19 irrelevant studies. After full-text reviewing, we excluded 15 studies, including 1 erratum paper, 2 reviews, 4 conference abstracts, and 1 ex-vivo study. In addition, we excluded 2 studies that did not use TE, 4 studies that did not mention our outcomes, and 1 study that was missing data. There were two publications that came from one group of authors, conducting the same protocol and reporting the same outcome in one year. We considered them as one study, therefore reported one study and excluded the other. Besides, the studies that came from the same group of authors and conducting the same protocol, but reported different outcomes were considered as separated publications to discuss. Finally, ten publications were included in the current systematic review. No additional articles were found by reading the reference lists of the included studies.

### 3.2. Study Characteristics

*Types of PD models:* rats and mice were used with two models, including the 1-methyl-4-phenyl-1,2,3,6-tetrahydropyridine (MPTP) model (n = 6) and the 6-hydroxydopamine (6-ODHA) model (n = 4). Eight studies used young animals (7–8 weeks old), whereas two studies used middle-aged animals (6–10 months old) (Table 1).

*Type of TE training protocol:* there were three types of TE protocol: preventive training (n = 2), treatment training (n = 6), and both preventive and treatment training (n = 2). The exercise duration ranged from 1 to 18 weeks. TE training was conducted 3–7 days/week, 20–60 min for each session. The speed of the TE ranged between 10 m/min to 21 m/min (Table 1).

*Type of outcome:* the included studies analyzed substantia nigra (n = 7), striatum (n = 7), and hippocampus (n = 1). For the electron transport system outcome, six studies reported the expression of neural mitochondrial complex I–V and cytochrome *c*. One study reported the ATP production outcome. For the mitochondrial biogenesis outcome, six studies evaluated the levels of neural mitochondrial biogenesis regulators, including sirtuin-3 (SIRT3), sirtuin-1 (SIRT1), AMP-activated protein kinase (AMPK), peroxisome proliferator-activated receptor gamma coactivator 1-alpha (PGC-1α), Nuclear respiratory factor 1 and 2 (NRF-1,2), and mitochondrial transcription factor A (TFAM). Two studies reported the alterations of import machinery, including translocase of the outer membrane 20 and 40 (TOM-20, TOM-40), translocase of the inner membrane-23 (TIM-23), and mitochondrial heat shock protein (mtHSP70). For the mitochondrial dynamic outcome, two studies reported fusion proteins, i.e., dynamin-like 120 kDa protein (OPA-1) and mitofusin-1,2 (MFN-1,2), as well as fission proteins, i.e., dynamin-related protein-1 (Drp-1) and the phosphorylation at Ser637 of Drp-1 (Drp-1^Ser637^). For the mitophagy outcome, three studies reported the dysfunctional mitochondria detector proteins, i.e., PTEN-induced kinase-1 (PINK1), parkin, and p62; autophagosomal proteins, i.e., beclin-1 and microtubule-associated protein 1A/1B-light chain 3 (LC3II); as well as lysosomal proteins, i.e., lysosome-associated membrane proteins 2 (LAMP2) and cathepsin L (Table 1).

### 3.3. Outcome Summary

#### 3.3.1. Effects of TE Training on Neural Mitochondrial Respiratory Deficiency in PD

Three studies analyzed the effects of TE training on mitochondrial complex I in PD [15,23,26]. Two of those showed that the protein levels of complex I were reduced in PD compared to normal, whereas TE training enhanced their levels in PD [15,26]. However, the other study observed that the protein levels of complex I were similar among the normal control group, the PD group, and the TE-trained PD group [23].

Two studies observed that the protein levels of cytochrome *c* in neural mitochondria were reduced in PD compared to normal, whereas TE training increased those levels in PD [20,24]. One study reported that ATP production was reduced in PD compared to normal, whereas TE training enhanced ATP production in PD [25].

Five authors accessed the expression of complexes II, III, IV, and V, reporting different results [15,20,21,23,26]. One study reported that the protein levels of complexes II, III, IV, and V were unchanged among the normal control group, the sedentary PD group, and the TE-trained PD group [26]. The other study showed that the protein levels of complex II and complex V were reduced in PD compared to normal and those levels were restored by TE training in PD, whereas the protein levels of complex III and complex IV were unchanged among the normal control group, the PD group, and the TE-trained PD group [23]. Another study observed that TE training reduced the overexpression of complex II, complex III, and complex IV protein levels in PD, whereas complex V protein levels were unchanged among the normal control group, the PD group, and the TE trained-PD group [15]. Two studies showed that the protein levels of complex IV were reduced in PD compared to normal, whereas TE training increased those levels in PD [20,21].

#### 3.3.2. Effects of TE Training on Neural Mitochondrial Biogenesis in PD

Six publications analyzed TE effects on biogenesis regulators of neural mitochondria in PD [16,20,22,23,24,26]. Four of those showed that the protein levels of biogenesis regulators, including SIRT3 [23], SIRT1 [16,20], PGC-1α [20,26], NRF-1,2 [20,23,26], and TFAM [20,23,26] were reduced in PD compared to normal, whereas TE training increased those levels in PD. In the other study that analyzed mRNA and protein levels of biogenesis regulators, they observed reduced levels of two biogenesis regulators (AMPK and PGC-1α) along with increased levels of two others (SIRT1 and TFAM) in PD compared to normal [22]. However, in this study, all of those levels were enhanced by TE training in PD [22]. On the contrary, another study showed that the mRNA levels of biogenesis regulators (PGC-1α and TFAM) were increased in PD compared to normal, and those levels were reduced by TE training in PD [24].

Two studies analyzed the effects of TE on translocase factors of neural mitochondria in PD [15,21]. One study reported that the protein levels of translocase proteins (TOM-20, TOM-40, TIM-23, and mtHSP70) were reduced in PD compared to normal, whereas TE training increased those levels in PD [21]. The other study showed that the level of translocase protein (TOM-20) in the substantia nigra was reduced in PD and recovered by TE training, but its level in the striatum was similar among the normal control group, the PD group and the TE-trained PD group [15].

#### 3.3.3. Effects of TE Training on Neural Mitochondrial Dynamics in PD

Two studies analyzed the effects of TE training on mitochondrial fusion and fission proteins [15,23]. They reported that the protein levels of fusion proteins (OPA1, MFN2) were reduced in PD compared to normal, whereas TE training enhanced those levels in PD [15,23]. Regarding neural mitochondrial fission, one of two studies showed that the fission protein (Drp-1) was reduced in PD compared to normal, whereas TE training enhanced those levels in PD [15]. However, the other study showed that the anti-fission protein level (p-Drp1^Ser637^) was reduced in PD compared to normal, whereas TE training enhanced those levels in PD [23].

#### 3.3.4. Effects of TE Training on Neural Mitophagy in PD

Three studies analyzed the effects of TE training on neural mitophagy in PD [15,20,27]. Those studies showed that the levels of mitophagy detector proteins, including PINK1 [15,27], parkin [27], and p62 [20,27] were increased in PD compared to normal, whereas TE training reduced those levels in PD. Two of those studies showed that the levels of autophagosomal proteins, including beclin-1 [20] and LC3 II/I [20,27] were increased in PD compared to normal, whereas TE training had no effect on their levels in PD. One of those studies reported that the levels of lysosomal proteins (LAMP2 and cathepsin L) were reduced in PD compared to normal, whereas TE training enhanced those levels in PD [27].

### 3.4. Study Quality Evaluation

All of the studies were published in peer-reviewed journals (item 1), providing a statement of compliance with regulatory requirements (item 9). All of the studies used validated models of PD (item 7). However, 100% of the studies did not mention allocation concealment (item 4), blinded assessment (item 5), or sample size calculation (item 8). Five papers (50%) did not clearly explain the anesthetics process (item 6). Four studies (40%) did not report the randomization of allocation (item 3). Three studies (30%) did not provide temperature control (item 2). Two studies (20%) did not provide conflicts of interest statements (item 10). Together, according to the CAMARADES checklist, the median quality score was 5.7/10 (Table 2).

## 4. Discussion

### 4.1. Summary of Evidence

**Figure 2:**
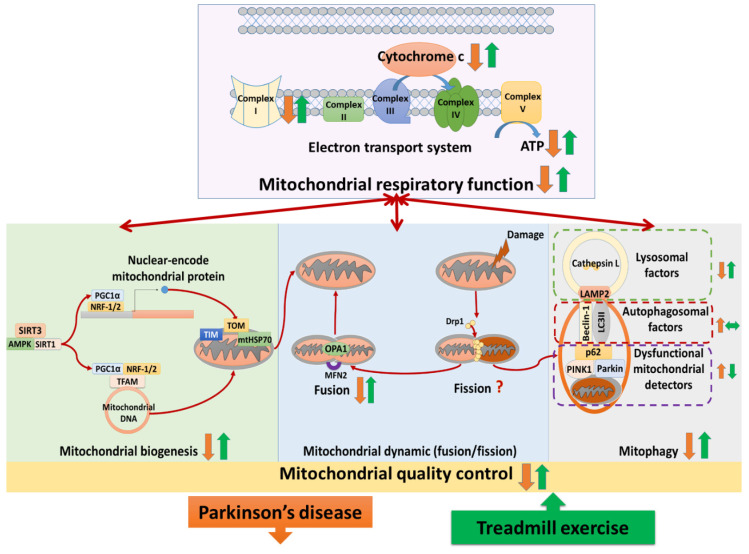
The hypothesized figure. The figure shows the structure of the electron transport system and the cycle of mitochondrial quality-control. The electron transport system includes complexes I–V and cytochrome *c*, taking the primary responsibility for producing ATP (adenosine triphosphate) in neurons. Neural mitochondrial quality-control is the balance among mitochondrial biogenesis, mitochondrial dynamics (fusion/fission), and mitophagy (autophagy of mitochondria). Mitochondrial biogenesis produces the new mitochondria and mitochondrial content, controlled by biogenesis regulators (e.g., SIRT3, SIRT1, AMPK, PGC-1α, NRF-1,2, and TFAM). SIRT3/SIRT1/AMPK activates PGC-1α, then PGC-1α binds with NRF-1,2 in both the nucleus and mitochondria. In the nucleus, PGC-1α and NRF-1,2 promote the production of nuclear-encoded mitochondrial proteins. Nuclear-encoded mitochondrial proteins are imported into mitochondria, which involves the import machinery (e.g., TOM, TIM, and mtHSP70). In the mitochondria, PGC-1α and NRF-1,2 bind with TFAM to activate replication, transcription, and translation of mitochondrial DNA. Mitochondrial fusion merged mitochondria to the large mitochondrion, regulated by OPA-1 in the inner membrane and MFN-2 in the outer membrane. When the mitochondria are damaged, Drp-1 promotes mitochondrial fission to segregate and provide dysfunctional mitochondria for the mitophagy process to destroy. In the mitophagy process, the overexpression of detectors (e.g., PINK1, parkin, p62) on the dysfunctional mitochondrial membrane recruits autophagosomal factors (e.g., beclin-1 and LC3II) to form an autophagosome. Supported by LAMP2, the autophagosome fuses with lysosomes, destroying the dysfunctional mitochondria by enzymes (e.g., cathepsin L). In Parkinson’s disease, the evidence shows that complex I, cytochrome *c,* and ATP production in the mitochondria are reduced. Moreover, the mitochondria quality-control is dysregulated in Parkinson’s diseases, characterized by biogenesis reduction, fusion/fission imbalance, and mitophagy reduction. The included studies suggested that treadmill training activated complex I, cytochrome *c*, and ATP production. Additionally, treadmill training was shown to optimize the levels of mitochondrial biogenesis regulators (SIRT3, SIRT1, AMPK, PGC-1α, NRF-1,2, and TFAM), translocase factors (TOM-20, TOM-40, TIM-23, and mtHSP70), fusion proteins (OPA-1 and MFN-2), and lysosomal factors (LAMP2 and cathepsin L) as well as reducing dysfunctional mitochondrial detectors (PINK1, parkin, and p62). These data imply that treadmill training could attenuate neural mitochondrial respiratory deficiencies and neural mitochondrial quality-control dysregulation in Parkinson’s disease.

Our review findings are synthesized as follows: (1) Treadmill training attenuated neural mitochondrial respiratory deficiency in Parkinson’s disease, supported by the evidence that treadmill training normalized the levels of complexes I–V, cytochrome *c*, and ATP production in the Parkinsonian brain. (2) Treadmill training optimized neural mitochondrial biogenesis in Parkinson’s disease, supported by the evidence that treadmill training increased or normalized the levels of biogenesis regulators (SIRT3, SIRT1, AMPK, PGC-1α, NRF-1,2, and TFAM) and import machinery (TOM-20, TOM-40, TIM-23, and mtHSP70) in the Parkinsonian brain. (3) Treadmill training enhanced the neural mitochondrial fusion in Parkinson’s disease, supported by the evidence that treadmill training increased mitochondrial fusion factors (OPA-1 and MFN-2) in the Parkinsonian brain. (4) Treadmill training repaired the impairment of mitophagy in Parkinson’s disease, supported by the evidence that treadmill training reduced the levels of dysfunctional mitochondria detectors (PINK1, parkin, and p62) and increased the levels of lysosomal factors (LAMP2 and cathepsin L) in the Parkinsonian brain. Taking these findings with the previously hypothesized pathophysiology of Parkinson’s disease together, we drew a hypothesized figure (Figure 2), which suggests that treadmill training could counteract the neurodegeneration of Parkinson’s disease in both the neural mitochondrial respiratory system and neural mitochondrial quality-control. Our review findings implied that treadmill training might provide therapeutic effects to slow down the progression of Parkinson’s disease.

As mentioned, the neurodegeneration of Parkinson’s disease on the neural mitochondrial respiratory system is characterized by a complex I deficit, cytochrome *c* release, and ATP depletion [6]. The included studies showed that treadmill training enhanced the complex I level, cytochrome *c* concentration, and ATP production in Parkinsonian neural mitochondria [15,20,24,25,26], suggesting that treadmill training could attenuate the neural mitochondrial respiratory deficiency in Parkinson’s disease. Supportively, previous evidence showed that treadmill training increased brain-derived neurotrophic factor (BDNF), which activated neural mitochondrial complex I in Parkinson’s disease [28,29]. Of note, a mitochondrial complex I deficit induces oxidative stress and cytochrome *c* release, promoting neural apoptosis in Parkinson’s disease [4,7,30]. Treadmill training appeared to enhance BDNF and complex I level to reduce the progressive development of Parkinson’s disease.

In the current included studies, the abnormal expression of complex II, complex III, complex IV, and complex V in Parkinson’s disease were varied [15,20,21,23,26]. Consistent with those data, previous studies showed that the alterations of complexes II–V in Parkinson’s disease were diverse, which seemed to be remodeled in response to the complex I deficits [5,11,30]. The current included studies showed that treadmill training normalized the levels of neural mitochondrial complexes II–V, suggesting that treadmill training prevented the abnormal remodeling of complexes II–V in Parkinson’s disease. In addition to Parkinson’s disease, a previous study showed that 12-week treadmill training attenuated the deficits of neural mitochondrial complexes I–IV in the brain of a Huntington’s disease mice model [31]. Another study reported that 36-week treadmill training normalized the protein levels of complexes I–V in the cerebral cortex and hippocampus of 40-week-old rats compared to 5-week-old rats [32]. Therefore, we further hypothesized that treadmill training could repair the impairment of the electron transport system in neurodegenerative disorders.

In the physiologic condition, mitochondrial biogenesis is regulated to optimize the neural energy status and guarantee neuronal survival [9]. In Parkinson’s disease, α-synuclein binds with genes to inhibit the expression of mitochondrial biogenesis regulators in the brain [33]. The current included studies showed that treadmill training increased or normalized the levels of biogenesis regulators (SIRT3, SIRT1, AMPK, PGC-1α, NRF-1,2, and TFAM), as well as the import machinery (TOM-20, TOM-40, TIM-23, and mtHSP70) [16,20,22,23,24,26], suggesting that treadmill training could optimize neural mitochondria biogenesis to attenuate the neural energy deficits in Parkinson’s disease.

One theory explained that exercise increased oxygen consumption, promoting metabolism challenge and mitochondrial adaptive responses, and thus, the recovery of neural mitochondrial biogenesis [34]. Another researcher suggested that treadmill training may reduce α-synuclein accumulation in Parkinson’s disease, thereby increasing the translation of biogenesis regulators and the import of nuclear-encoded mitochondrial proteins into mitochondria [20]. Therefore, our review findings suggested that treadmill training could improve neural mitochondrial biogenesis in Parkinson’s disease. Additionally, one study showed that 12-week treadmill training also increased the protein levels of biogenesis regulators (SIRT1, PGC-1α, and TFAM) along with the reduction of beta-amyloid accumulation in the brain of Alzheimer’s disease rats model [35]. Another study reported that 12-week treadmill training enhanced neural mitochondrial biogenesis in a Huntington’s disease mice model, as evidenced by increases in the ratio of mitochondrial DNA/nucleus DNA in the brain [31]. Overall, we further hypothesized that treadmill training could improve neural mitochondrial biogenesis in neurodegenerative disorders.

In the current review, we found that treadmill training activated neural mitochondrial fusion in Parkinson’s disease, as evidenced by increases of mitochondrial fusion factors (OPA-1 and MNF-2) after treadmill training in the Parkinsonian brain [15,23]. Supportively, a previous study showed that 12-week treadmill training increased the levels of fusion proteins (OPA-1 and MNF-2) in the hippocampus of an Alzheimer’s disease mouse model [36]. The improvement of neural mitochondrial fusion could reduce the genome mutations and protect the healthy neural mitochondria against the neurodegenerative progression.

Regarding neural mitochondria fission, two included studies reported opposite findings [15,23]. One study showed that neural mitochondrial fission was increased in PD and reduced by TE training [23]. In contrast, another showed that neural mitochondrial fission was reduced in PD and restored to normal by TE [15]. One possible explanation for such a discrepancy is that neural mitochondrial fission is controlled by neural optimization and homeostasis, which are different among Parkinson’s disease models [23]. Supportively, a previous study reported that 12-week treadmill exercise normalized the protein levels of fission factors (Drp-1) in the hippocampus of Alzheimer’s disease mice model [36]. However, due to the lack of studies, the effect of treadmill training on neural mitochondrial fission was not provided by the current systematic review.

In addition, our review found that treadmill training restored the mitophagy process in Parkinson’s disease. In the included studies, treadmill training reduced mitophagy detectors (PINK1, parkin, and p62) but there was no change in autophagosomal factors (beclin-1 and LC3II) in Parkinson’s disease [15,20,27], suggesting that treadmill training reduced the accumulation of dysfunctional mitochondria as well as maintained autophagosome flux in Parkinson’s disease. Moreover, treadmill training has been shown to upregulate lysosomal proteins (LAMP2 and cathepsin L) in Parkinson’s disease, implying that treadmill training increased the activities of the lysosome to fuse with the autophagosome and destroy dysfunctional mitochondria in neural cells [27]. The underlying mechanisms of those benefits may be associated with treadmill training-reduced α-synuclein aggregation in the Parkinsonian brain [20,27]. In addition to Parkinson’s disease, a previous study reported that 12-week treadmill training also enhanced mitophagy in an Alzheimer’s disease mice model, as evidenced by reductions in the levels of dysfunctional mitochondrial detectors (PINK1 and p62) [37]. Thus, we hypothesized that treadmill training prevents the progressive development of neurodegenerative disorders partially through improving neural mitophagy.

### 4.2. Study Quality Evaluation

In the current systematic review, we used the CAMARADES checklist to evaluate the methodological quality of the included studies strictly. Because the included studies conducted treadmill training on animals, it is not possible to allocate them in a blinded manner. As is common in animal studies, none of the included studies calculated sample size or used blinded outcome measurements. However, most of the included studies randomly allocated experimental groups and declared no conflicts of interest, suggesting that those studies had no or only minor selection bias and reporting bias. Furthermore, all of the included studies were published in peer-reviewed journals—credible resources. Although there were two publications that were from one group of authors and conducted the similar protocol, they conducted with different sample sizes and reported different aspects of neural mitochondrial functions [24,25]. Similarly, the other two publications were from another group of authors and had the similar protocol, but provided different outcomes [20,21]. Therefore, the data analysis issues (or reporting bias) in those studies might be considered as moderate concern. Taken together, the reviewed findings from the included studies are reasonable. However, due to a lack of data and some concerns mentioned above, further studies need to be conducted to support our review findings.

### 4.3. Limitations

There were several limitations in our systematic review. First, we only collected the English-published articles with full text, and therefore, we did not include the evidence from non-English articles, conference articles, unpublished articles, or locally published articles. Second, most of the included studies used young animals (n = 8), and all of the included studies used toxin models, but not genetic models. Furthermore, eight of ten studies used male animal models, but not female animal models. All those biases mentioned above limited the generalizability of our review findings to the entire Parkinson’s disease population. Third, the studies reviewed herein used treadmill training with various duration (1–18 weeks) and intensities (10–21 m/min). Most of the included studies (n = 9) did not compare durations or intensities of treadmill training on neural mitochondrial functions in Parkinson’s disease. Therefore, the current systematic review cannot provide evidence for the optimal protocol of treadmill training in Parkinson’s disease. Finally, it should be noted that neural mitochondrial dysfunction in Parkinson’s disease involves many factors such as oxidative stress, neuro-inflammation, α-synuclein accumulation, and calcium flux [4]. Our systematic review reported the therapeutic effects of treadmill training on neural mitochondrial functions in Parkinson’s disease but cannot provide cause-effects of why treadmill training could attenuate neural mitochondrial respiratory deficiency and neural mitochondrial quality-control dysregulation in Parkinson’s disease.

### 4.4. The Implications for Future Research

In the studies reviewed here, treadmill training appeared to improve both symptoms and neural mitochondrial function in Parkinson’s disease animal models. An included study showed that, alongside the enhancement of mitochondrial functions, stride length and swing speed in Parkinsonian rats were enhanced after one month of treadmill training [15]. The other included study reported that, along with neural mitochondrial functions improvement, rest tremor and rigidity in Parkinsonian mice were reduced after 6 weeks of treadmill training [23]. Those results suggested that there may be a possible correlation between symptoms and neural mitochondrial function in Parkinson’s disease. Supportively, in clinical observations, a previous randomized controlled clinical trial showed that treadmill training improved symptoms and quality of life in Parkinson’s disease patients [38]. Moreover, a previous systematic review showed that treadmill training improved the cognitive function of Parkinson’s disease patients [39]. Thus, we hypothesized that treadmill training might reduce the motor and cognitive impairments in Parkinson’s disease patients partially through improving neural mitochondrial functions. Further clinical studies should address the correlation between symptoms and mitochondrial function in Parkinson’s disease patients with treadmill training.

As mentioned, there were two included studies showed that treadmill exercise training before the onset of PD might also maintain neural mitochondrial functions in animal models of PD [16,22]. Consistently, a previous study provided that 8 weeks of treadmill training could enhance neural mitochondrial biogenesis regulator (PGC-1α protein) as well as neural mitochondrial DNA in the hippocampus of the healthy aged mice (21-month-old) [40]. Another study showed that long-term treadmill training (36 weeks) could increase the protein expressions of neural mitochondrial electron transport chain (complex I, III, and IV) as well as neural mitochondrial biogenesis (SIRT-1, PGC-1α, AMPK) in the hippocampus of 42-week-old healthy rats [32]. Those findings implied that treadmill training might control both neural mitochondrial respiratory functions and neural mitochondrial quality-control to prevent or attenuate the damage of neurodegeneration, including PD. Supportively, regarding clinical evidence, a previous systematic review and meta-analysis has been shown that exercise training could reduce the risk of PD [41]. Therefore, people with the high-risk of neurodegeneration or the neurodegenerative patients should devote themselves to exercise training with treadmill exercise as one choice.

The animal models of PD used in the included studies, including MPTP model and 6-ODHA model, have been proven to be the reliable models and are commonly used to mimic PD in animals [42]. In MPTP model, once entering brain blood barrier, MPTP directly inhibit neural mitochondrial complex I, thereby reduce neural mitochondrial respiration and damage neural mitochondrial functions [42,43]. MPTP has been proven to induce oxidative stress (the accumulation of MPP+ could produce reactive oxygen species) and neuroinflammation (MPTP could activate macrophages, microglia) [43]. In 6-ODHA model, previous evidence proved that 6-ODHA not only generate reactive oxygen species, but also interact with mitochondrial complex I and complex IV to inhibit neural mitochondrial respiration [42]. It should be noted that, in PD, both neural oxidative stress and neuroinflammation could further promote neural mitochondrial dysfunction [44,45]. Alongside the improvement of neural mitochondrial functions, the results of the included studies showed that treadmill training could enhance the levels of antioxidants (e.g., superoxide dismutase) in both neural cytoplasm and neural mitochondria in MPTP mice [20,25] as well as reduce the levels of pro-inflammatory cytokines, including TNF-α, IFN-γ, and IL-1β level in the striatum and hippocampus of 6-ODHA mice [16]. Supportively, a previous study showed that 14-day treadmill training reduced neural oxidative stress in PD, as evidenced by the decreased levels of lipid peroxidation in the striatum of 6-ODHA rats [46]. Another study provided that 4-week treadmill training could attenuate neuroinflammation via the inactivation of microglia in MPTP mice [47]. Those findings might suggest that treadmill exercise could protect neural mitochondrial functions due partially to attenuating the damage of neural oxidative stress and neuroinflammation on neural mitochondria in PD. This issue needs to be addressed in the further studies to clarify the complicated interdependences among the mechanisms of neurodegenerative disorders, including PD.

Epidemiological statistics showed that men have higher risks and proportion to suffer from Parkinson’s disease than women in all ages [48]. This phenomenon might be also due partially to the gender-specific characteristics of neural mitochondria. Evidence indicated that the activities of neural mitochondrial electron transport system (complex I–V) and neural mitochondrial capacities in males was lower than that in females [49]. Moreover, the dysregulation of genes associated to neural mitochondrial respiration (neural oxidative phosphorylation) has been proven to be stronger in males when compared to females in PD, suggesting that neural mitochondria in males are more sensitive to the damage of several Parkinsonian mechanisms (e.g., neuroinflammation, neural oxidative stress) than that in females [48]. Supportively, another study provided that estradiol (a hormone in females) could restore the number of hippocampal mitochondria in aged rats (24-month-old), implying that estradiol could maintain neural mitochondrial biogenesis in females [50]. Basing on the differences in neural mitochondrial functions between two genders mentioned here, we might hypothesize that the effects of the same protocol of treadmill training in neural mitochondrial respiratory function as well as neural mitochondrial quality-control in two genders might be different. Therefore, in order to further understand the mitochondria-related effects of treadmill exercise as well as to find the suitable protocol of treadmill training for each gender in PD, further studies are required to evaluate and compare the benefits of treadmill exercise on neural mitochondrial functions in both males and females, regarding the underlying mechanisms (e.g., genes, hormones).

Because treadmill exercise is widely applied for symptomatic improvement in patients with Parkinson’s disease [38], the highlighted issue for future clinical trials is how to determine the optimal protocol of treadmill training, which could slow down the progression and improve symptoms in Parkinson’s disease patients. To establish this issue, it is necessary to have an efficient method to measure the change of Parkinson’s disease mechanisms during and after treadmill training. Recently, the evidence has suggested that 31 phosphorus-magnetic resonance spectroscopy (^31^P-MRS) is a promised approach for the evaluation of mitochondrial function in Parkinson’s disease patients in both the rest-stage and moving-stage [5]. Although the application of ^31^P-MRS on Parkinson’s disease needs to be further investigated, future studies should determine the optimal protocol of treadmill training on neural mitochondrial function in Parkinson’s disease by carrying out TE training on Parkinson’s disease patients with a ^31^P-MRS monitor.

## 5. Conclusions

Our systematic review summarized the recent evidence from animal studies to determine the effects of treadmill training on the neural mitochondrial respiratory deficiency and neural mitochondrial quality-control dysregulation in Parkinson’s disease. From the included studies with various Parkinson’s disease models and treadmill training protocols, both preventive treadmill training and treatment training were shown to positively affect neural mitochondria in the Parkinsonian brain. Overall, the review found that treadmill training could attenuate the abnormalities of neural mitochondrial complexes I–V, cytochrome *c*, and ATP production, as well as improve the neural mitochondrial biogenesis, neural mitochondrial fusion, and neural mitophagy in Parkinson’s disease. Our systematic review suggested that treadmill training might attenuate the neurodegeneration of Parkinson’s disease on neural mitochondria, leading to prevention of or delaying the development of Parkinson’s disease.

Further interdisciplinary studies are required to investigate the effects of treadmill training on the neural mitochondrial respiratory system, biogenesis, dynamics, and mitophagy in both genetic models and toxin models of Parkinson’s disease. Additionally, clinical studies should clarify the possible therapeutic applications through different exercise interventions into neural mitochondrial dysfunction in Parkinson’s disease.

## Figures and Tables

**Table 1:** Characteristics and outcomes of the included studies.

Study	Model	Treadmill Exercise	Brain’s Tissue	Outcomes			
				Mitochondrial Respiratory Function	Mitochondrial Biogenesis	Mitochondrial Dynamic	Mitophagy
Koo and Cho, 2017 [20]	MPTP model on male mice (7-wk-old), induced by 25 mg/kg MPTP i.p, twice/wk for 5 wks	After PD induction. Duration: 40–60 min/day, 5 days/wk, 8 wks. Speed: 10–12 min/m.	Substantia Nigra Striatum	Compared to normal, protein levels of complex IV and cytochrome *c* were reduced in PD group. TE training enhanced those levels in PD.	Compared to normal, protein levels of SIRT1, PGC-1α, NRF-1, and TFAM were reduced in PD group. TE training enhanced those levels in PD.		Compared to normal, the protein levels of p62, beclin-1 levels, and LC3II/I ratio were increased in PD group. TE training reduced p62 levels, but unchanged beclin-1 and LC3II/I levels in PD.
Koo et al., 2017a [21]	MPTP model on male mice (7-wk-old) induced by 25 mg/kg MPTP i.p twice/wk for 5 wks	After PD induction. Duration: 40–60 min/day, 5 days/wk, 8 wks. Speed: 10–12 min/m.	Substantia Nigra Striatum	Compared to normal, protein levels of complex IV were reduced in PD group. TE training enhanced those levels in PD.	Compared to normal, protein levels of TOM-40, TOM-20, TIM-23, and mtHSP70 were reduced in PD group. TE training enhanced those levels in PD.		
Rezaee et al., 2019 [22]	6-OHDA model on male rats (8-wk-old) induced by 2 μg/μL injected to the right medial forebrain bundle	Before PD induction. Duration: 25–50 min/day, 5 days/wk, 16 wks. Speed: 15–21 m/min.	Striatum		Compared to normal, both mRNA expression and protein levels of AMPK and PGC-1α were reduced in PD group. Both mRNA and protein levels of SIRT1 and TFAM were increased in PD group. All of those levels were enhanced by TE in PD group.		
Chuang et al., 2017 [15]	6-OHDA model on female rats (8-wk-old) induced by 15 μg/μL injected to the ascending mesostriatal pathway	After PD induction. Duration: 30 min/day, 7 days/wk, 4 wks. Speed: 15 m/min.	Substantia Nigra Striatum	Compared to normal, protein levels of complex I was reduced, whereas complex II, III, IV protein levels were increased in PD. TE increased complex I levels and reduced complex II, III, IV levels in PD. Complex V protein levels were unchanged among three groups.	Compared to normal, protein levels of TOM-20 were reduced in PD group. TE training enhanced TOM-20 levels in PD in Substantia Nigra. TOM-20 level was unchanged among three groups in striatum.	Compared to normal, protein levels of OPA-1, MFN-2, and Drp1 were reduced in PD group. TE training enhanced those levels in PD.	Compared to normal, the protein levels of PINK1 were increased in PD group. TE training reduced those levels in PD
Jang et al., 2018 [23]	MPTP model on male mice (7-wk-old) induced by 25 mg/kg MPTP i.p daily, 7 days	After PD induction. Duration: 60 min/day, 5 days/wk, 6 wks. Speed: 12 m/min.	Substantia Nigra	Compared to normal, protein levels of complex II and V were reduced in PD group. TE training enhanced complex II and V levels in PD. Complex I, III, IV protein levels were unchanged among all groups.	Compared to normal, protein levels of TFAM, NRF-1, and SIRT3 were reduced in PD group. TE training enhanced those levels in PD. PGC-1α protein level was unchanged among three groups.	Compared to normal, protein levels of OPA-1, MFN-2, and p-Drp1^Ser637^ were reduced in PD group. TE training enhanced those levels in PD.	
Tuon et al., 2015 [16]	6-OHDA model on male mice (8-wk-old) induced by 2 μg/μL injected to the striatum	Before PD induction. Duration: 50 min/day, 3–4 days/wk, 8 wks Speed: 13–17 m/min.	Striatum Hippocampus		Compared to normal, protein levels of SIRT1 were reduced in PD group. TE training enhanced those levels in PD.		
Patki and Lau, 2011 [24]	MPTP model on male mice (6–10 month-old) induced by 15 mg/kg MPTP, 10 doses, s.c., 5 wks	Before and after PD induction. Duration: 40 min/day, 5 days/wk, 18 wks. Speed: 15 m/min.	Striatum	Compared to normal, cytochrome *c* protein level in mitochondria was reduced in PD group. TE training enhanced those levels in PD.	Compared to normal, the mRNA levels of TFAM, PGC-1α were increased in PD group. TE training reduced those levels in PD to normal.		
Lau et al., 2011 [25]	MPTP model on male mice (6–10 month-old) induced by 15 mg/kg MPTP, s.c., 10 doses, 5 wks	Before and after PD induction. Duration: 40 min/day, 5 days/wk, 18 wks. Speed: 15 m/min.	Substantia Nigra Striatum	Compared to normal, the mitochondrial respiration stage 3–4, as well as ATP production, were reduced in PD group. TE training enhanced those levels in PD			
Ferreira et al., 2020 [26]	6-OHDA model on male mice (2–3 month-old) induced by 6 μg/μL injected to the striatum	After PD induction Duration: 40 min/day, 3 days/wk, 1 or 4 wks Speed: 10 m/min.	Substantia Nigra Striatum	Compared to normal, the protein level of complex I was reduced in PD group. 4-week TE training enhanced complex I levels in PD. Complex II-V in substantia nigra and complex I-V levels in striatum were unchanged among all groups.	Compared to normal, the levels of PGC-1α, NRF-1, and TFAM were reduced in PD. Those levels significantly increased to normal after 4 weeks training in Substantia Nigra. Those levels in striatum were unchanged among all groups.		
Hwang et al., 2018 [27]	MPTP model on male mice (8 week-old) induced by 25 mg/kg MPTP, i.p, 10 doses, 5 wks	After PD induction. Duration: 20 min/day, 5 days/wk, 8 wks. Speed: 15 m/min.	Substantia Nigra				Compared to normal, the protein levels of PINK1, Parkin, p62, and LC3II/I ratio were increased in PD group. TE training reduced the levels of PINK1, parkin, and p62, but unchanged LC3II/I ratio in PD. Compared to normal, protein levels of LAMP2 and Cathepsin L were reduced in PD group. TE enhanced those levels in PD.

Note: TE = treadmill exercise; PD = Parkinson’s disease; MPTP = 1-methyl-4-phenyl-1,2,3,6-tetrahydropyridine; 6-OHDA = 6-Hydroxydopamine; COX-I, COX-IV = Cytochrome c oxidase subunit I & IV; PGC-1α = peroxisome proliferator-activated receptor gamma coactivator 1-alpha; NRF-1,2 = nuclear respiratory factor 1 and 2; TFAM = mitochondrial transcription factor A; SIRT3 = sirtuin-3; AMPK = AMP-activated protein kinase; SIRT1 = sirtuin-1; TOM = translocase of outer membrane; TIM = translocase of inner membrane; mtHSP70 = mitochondrial heat shock protein 70; PINK1 = PTEN-induced kinase-1; OPA1 = Dynamin-like 120 kDa protein; MFN = Mitofusin-2; Dpr1 = Dynamin-related protein-1; LAMP2 = Lysosome-associated membrane protein 2; i.p = intraperitoneal injection; s.c = subcutaneous injection.

**Table 2:** The quality of studies basing-on the CAMARADES checklist.

Study	CAMARADES Checklist of Study Quality										
	1	2	3	4	5	6	7	8	9	10	Total
Koo and Cho, 2017 [20]	√	√	√				√		√	√	6
Koo et al., 2017a [21]	√	√	√				√		√	√	6
Rezaee et al., 2019 [22]	√	√	√			√	√		√	√	7
Chuang et al., 2017 [15]	√	√	√			√	√		√	√	7
Jang et al., 2018 [23]	√	√	√				√		√	√	6
Tuon et al., 2015 [16]	√	√				√	√		√	√	7
Patki and Lau, 2011 [24]	√					√	√		√		4
Lau et al., 2011 [25]	√					√	√		√		4
Ferreira et al., 2020 [26]	√		√				√		√	√	5
Hwang et al., 2018 [27]	√	√					√		√	√	5

Note: (1) Publication in peer-reviewed journal, (2) Statement of control of temperature. (3) Randomization of treatment or control, (4) Allocation concealment, (5) Blinded assessment of outcome, (6) Avoidance of anesthetics with marked intrinsic properties, (7) Use of animals with PD, (8) sample size calculation (9) Statement of compliance with regulatory requirements, (10) Statement regarding possible conflict of interest.

## Data Availability

No new data were created or analyzed in this study. Data sharing is not applicable to this article.
